# Supplementary material for: Developing OCHROdb, a comprehensive quality checked database of open chromatin regions from sequencing data
Source: Sci Rep. 2023 May 18;13:8106. doi: 10.1038/s41598-022-26791-x (PMC10195780; doi:10.1038/s41598-022-26791-x)
Supplement: Supplementary file 1 — Supplementary Information. [file 41598_2022_26791_MOESM1_ESM.docx]

**SUPPLEMENTARY INFORMATION**

**Figure S1: Number of replicate samples per cell type.** For the majority of cell types (161/194), there are at least two replicates per cell type. Number of technical and biological replicates varies across cells.

**Figure S2: Verifying number of replication tests required to accurately identifying replicable DHS.** We performed replication tests multiple times and combined them to identify replicable DHS clusters. Area under the curve remains above 0.95 after nine replication tests, highlighting that using ten replication tests is adequate for accurate identification of replicable DHS.


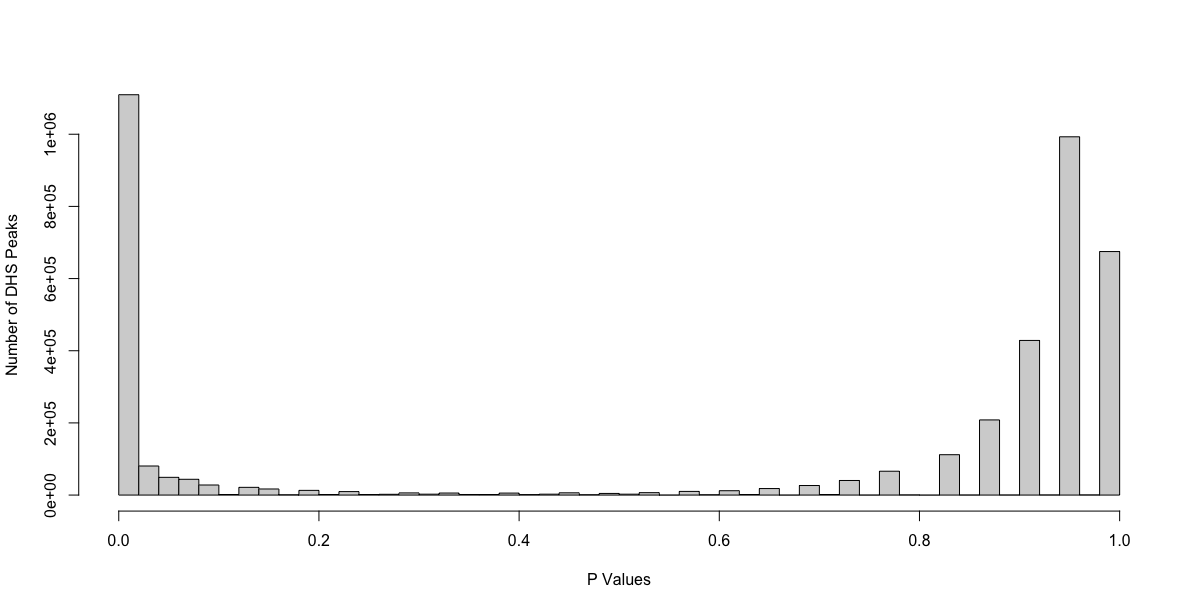


**Figure S3: Histogram of P values of DHS clusters.** 1,460,986 out of 4,020,940 DHS clusters (36.3%) passed the combined statistical test (P value of < 0.05).

**Figure S4: Partitioning heritability analysis of Multiple Sclerosis using all DHS sites and replicable DHS.** We applied partitioning heritability analysis on genome-wide association studies data from multiple sclerosis using both all DHS and replicable DHS. This analysis is based on LD score regression [[18]](https://paperpile.com/c/DxBA33/yP3Q0). Based on authors’ recommendations, the negative enrichment values should be ignored. In cell types where enrichment is positive, the differences between the heritability estimates are not significant and lie within the standard error of the estimates.

**Figure S5: Identifying replicable DHS overlapping a genomic region of interest and extracting information of each DHS.** Users can either download the full DHS data or (A) query their genomic region of interest and download region-specific replicable DHS. This will result in a file in Bed format containing genomic location and accessibility of each replicable DHS across 194 cell types. (B) In the interactive genome browser (Figure 4), users can click on a specific DHS and extract its information including its genomic location and accessibility levels across all cell types.

**TITLE OF SUPPLEMENTARY TABLE**

**Table S1: Information of 828 samples.** This table lists information of 828 samples, including the file accession ID in the ENCODE/Roadmap/GGR/Blueprint Project, biosample term name, biosample type, project, genome assembly and file download URL.
